# Extranasal epistaxis: a diagnostic challenge

**DOI:** 10.1590/S1808-86942012000200021

**Published:** 2015-10-20

**Authors:** Daniela Oliveira Teixeira, Luiz Carlos Alves de Oliveira, Enedir Borges Teixeira, Ana Maria Benvegnú, Letícia Moreira Flores Machado

**Affiliations:** a Radiology Resident, Specialized in Interventionist Vascular Radiology and Diagnostic/Therapeutic Neuroradiology, Physician at the Vascular Radiology Service of the Santa Maria University Hospital – HUSM; b ENT Resident, Physician at Dr. Oliveira Clinic; c Neurosurgery Resident, Physician at Hospital de Caridade Dr. Astrogildo de Azevedo – HCAA; d Medical Student at the Federal University of Santa Maria – UFSM, Intern at the Vascular Radiology Service; e Medical Student at the Federal University of Santa Maria – UFSM, Intern at the Vascular Radiology Service. Vascular Radiology Service (IRV) and Hospital de Caridade Dr. Astrogildo de Azevedo (HCAA)

**Keywords:** anemia, aneurysm, carotid artery, internal, epistaxis, stents

## INTRODUCTION

Epistaxis or nose bleeding is one of the most frequently seen emergency cases in the ENT practice. It may occur anteriorly or posteriorly, the latter being the more severe manifestation as it stems from branches of the sphenopalatine or ethmoidal arteries. Causes may be local (nose trauma, anatomic alterations, surgery, tumor) or systemic (vascular disorders, coagulopathy, drug therapy, infection, severe systemic disease, allergy).

Epistaxis originating from the intracranial internal carotid artery (ICA) is a rare condition¹. The most common cause for its occurrence is a ruptured aneurysm or pseudoaneurysm on the site². However, aneurysms in this portion of the ICA are even more rare and ruptures often manifest in conjunction with this type of bleeding^1^, ^2^, ^3^. Despite its low incidence rates, early diagnosis may prevent further complications and provide for better prognosis as therapy is promptly instituted^3^, ^4^.

## CASE DESCRIPTION

The patient is a Caucasian female, aged 28, with high blood pressure for 10 years. She has had a history of four months of profuse epistaxis to the left. Bleedings persist even after nasal packing. The patient reported she felt a throbbing sensation on her left ear before each episode.

She has been hospitalized numerous times due to bleeding, and has reported paresthesia and paresis on the left side of her body along with progressive left ear hearing loss. She has been administered 12 units of packed red blood cells. Her MRA and carotid angiography images were interpreted as normal. The patient also reported she underwent a septoplasty and a cauterization procedure at her hometown, but was unable to provide further details. Ten days after surgery she had a nasal-oral bleeding and was then referred to our service.

**Figure 1:**
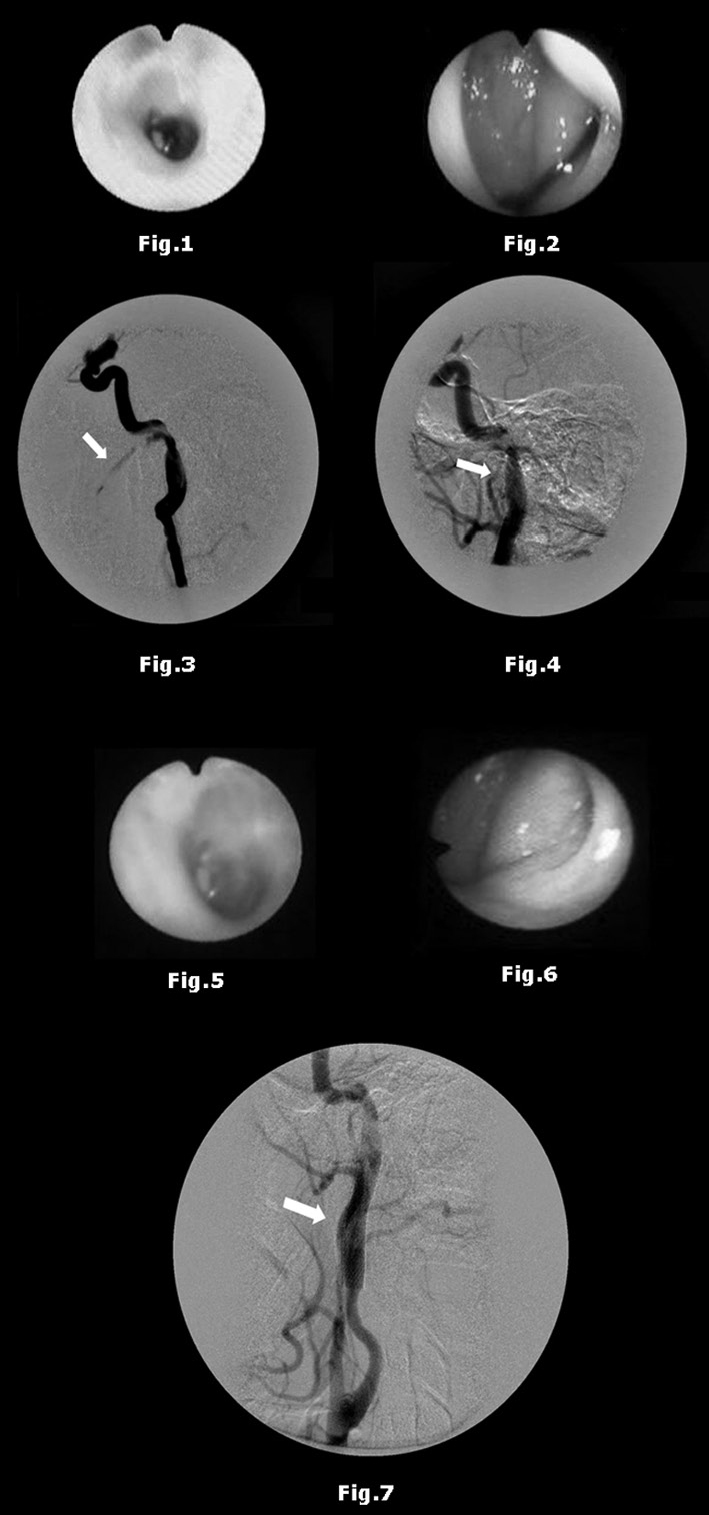
Ear endoscopy – Hemotympanum.

**Figure 2:**
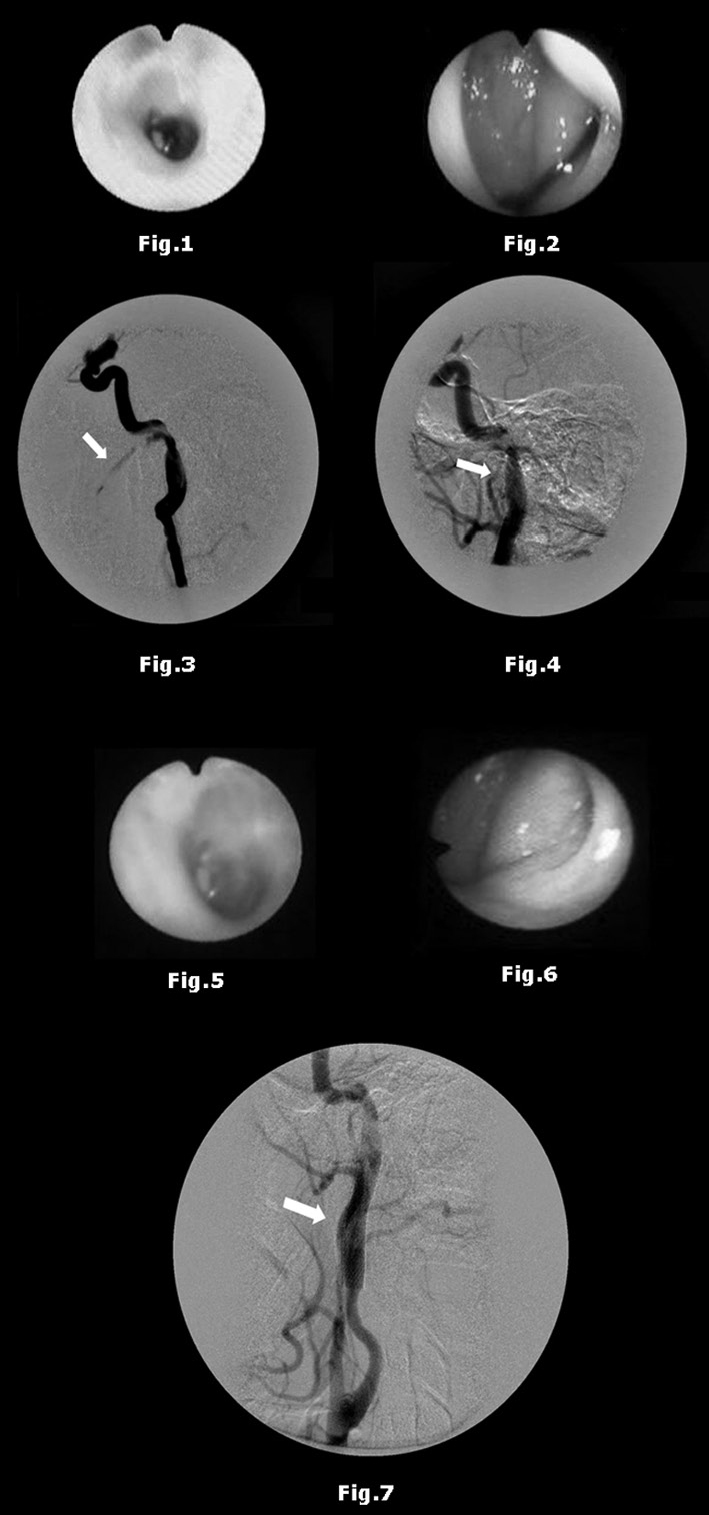
Nasal endoscopy – Blood flowing through the eustachian tube.

**Figure 3:**
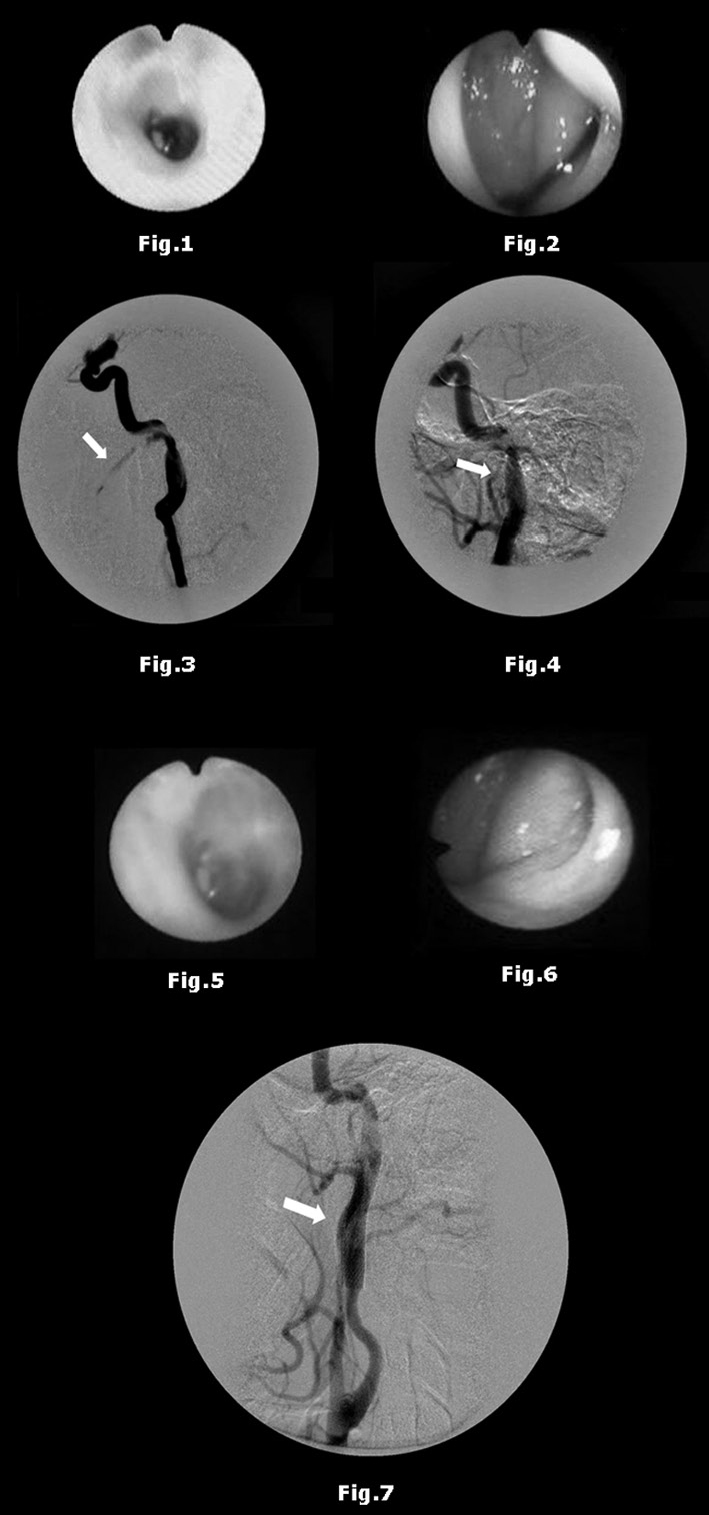
Cerebral angiogram before stenting – Image showing rupture on the petrous portion of the left ICA with contrast spilling over into the nasopharynx.

**Figure 4:**
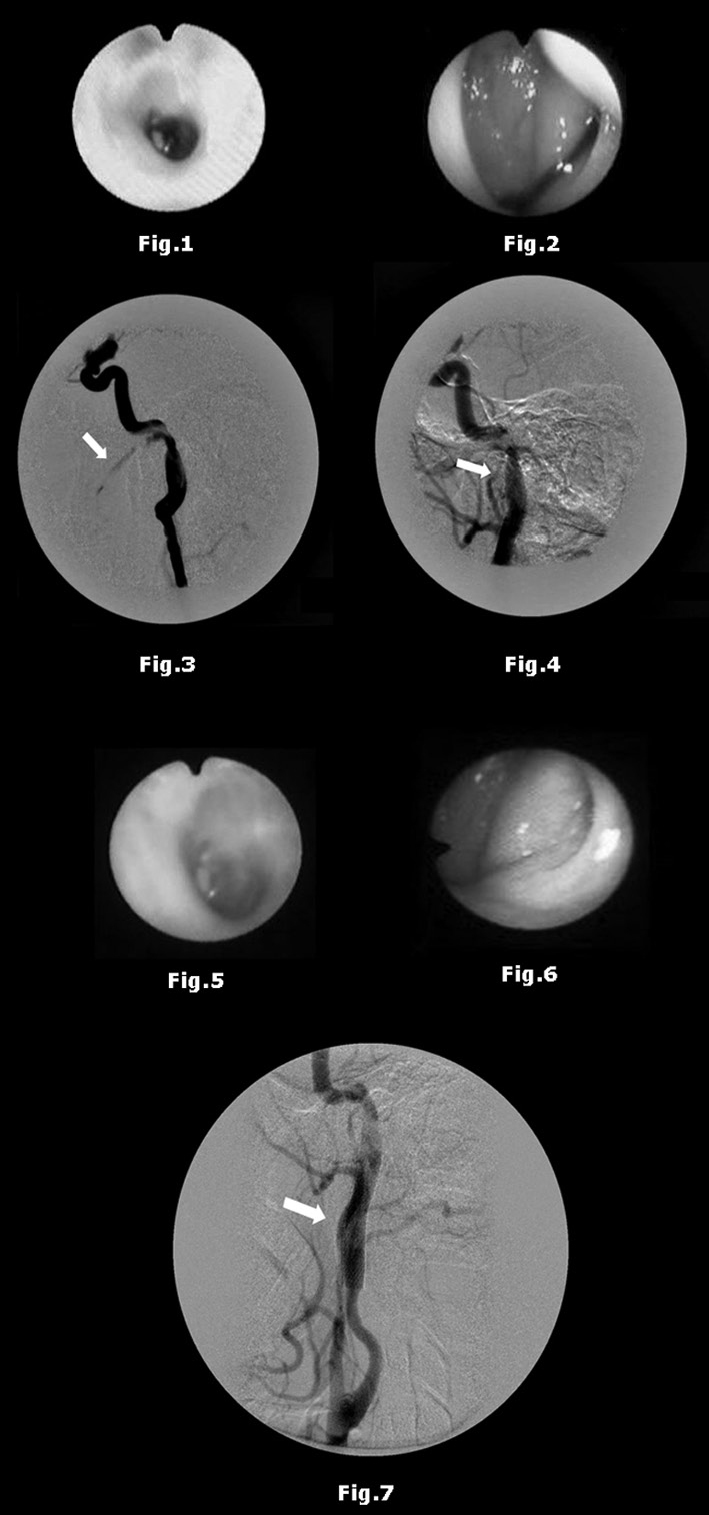
Cerebral angiogram after stenting – Absence of contrast spillover from left ICA into nasopharynx.

During initial examination the patient reported throbbing on her left ear, her hemoglobin was at 4g%, she had hemotympanum (Figure 1), alterations matching conduction dysacusis on hearing acuity tests, and blood flowing through the eustachian tube as seen on endoscopic examination (Figure 2). She was transfused immediately with PRBC and had an emergency cerebral angiogram done. The images showed a ruptured site on the petrous portion of the left ICA with contrast spilling over into the nasopharynx (Figure 3). A carotid occlusion test was carried out (the diseased artery was occluded with a balloon and contrast was injected in the contralateral carotid and vertebral arteries to verify communicating artery patency and the completeness of the circle of Willis). The test was negative, showing an incomplete circle of Willis (no blood passing through between the cerebral and cerebellar hemispheres). As the diagnosis was established, we opted for the placement of two percutaneous transluminal stents. During the procedure the patient had significant bleeding followed by limb paresthesia and paresis. She was given three units of PRBC. Immediate postoperative follow-up did not show any contrast spillover (Figure 4). The patient was referred to the neurology ICU. She had dysphagia for 24 hours, and presented left facial paresthesia and reduced left-side motor skills. She was discharged after staying in hospital for 12 days. She was still recovering from neurological deficit but was free from bleeding episodes. She was prescribed Clopidogrel 75mg/day.

**Figure 5:**
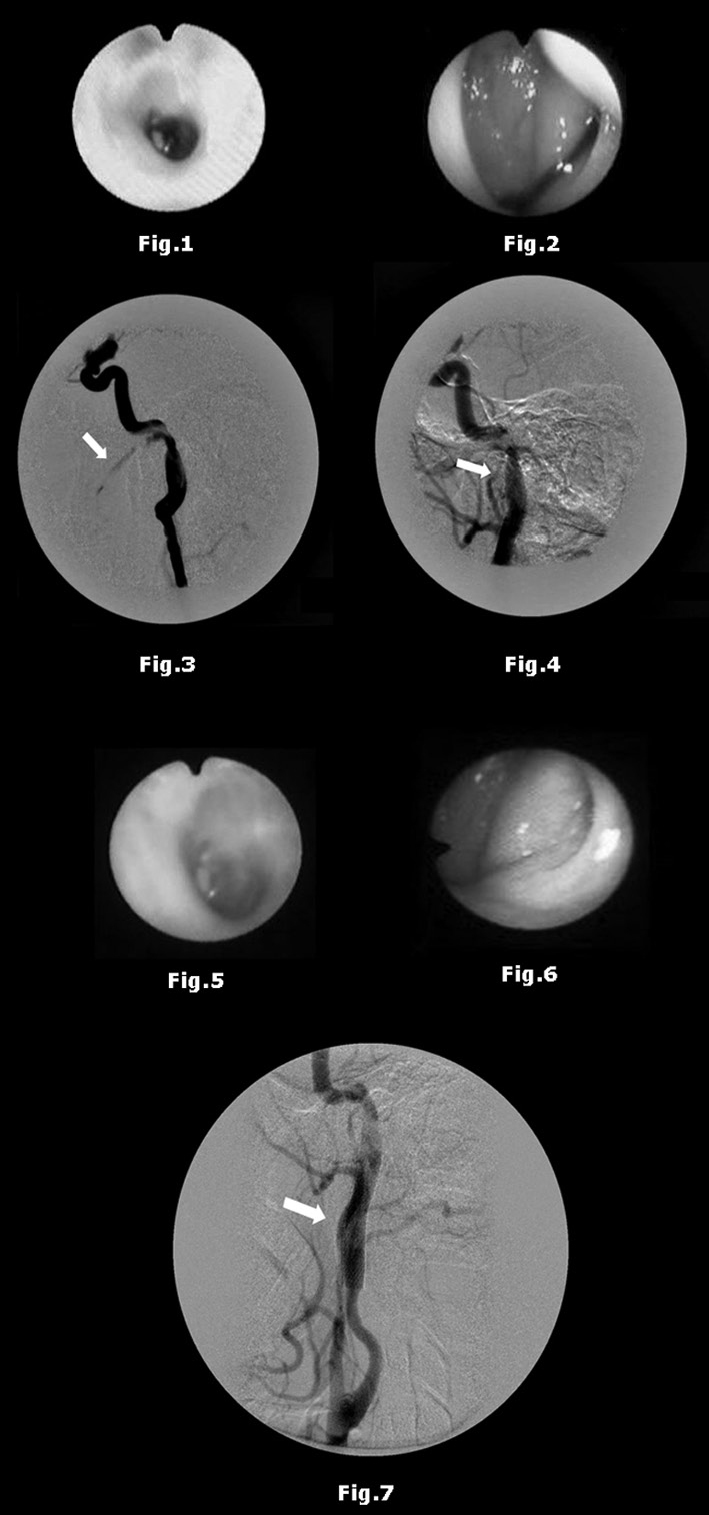
Ear endoscopy 45 days after stenting – nearly complete remission of hemotympanum.

**Figure 6:**
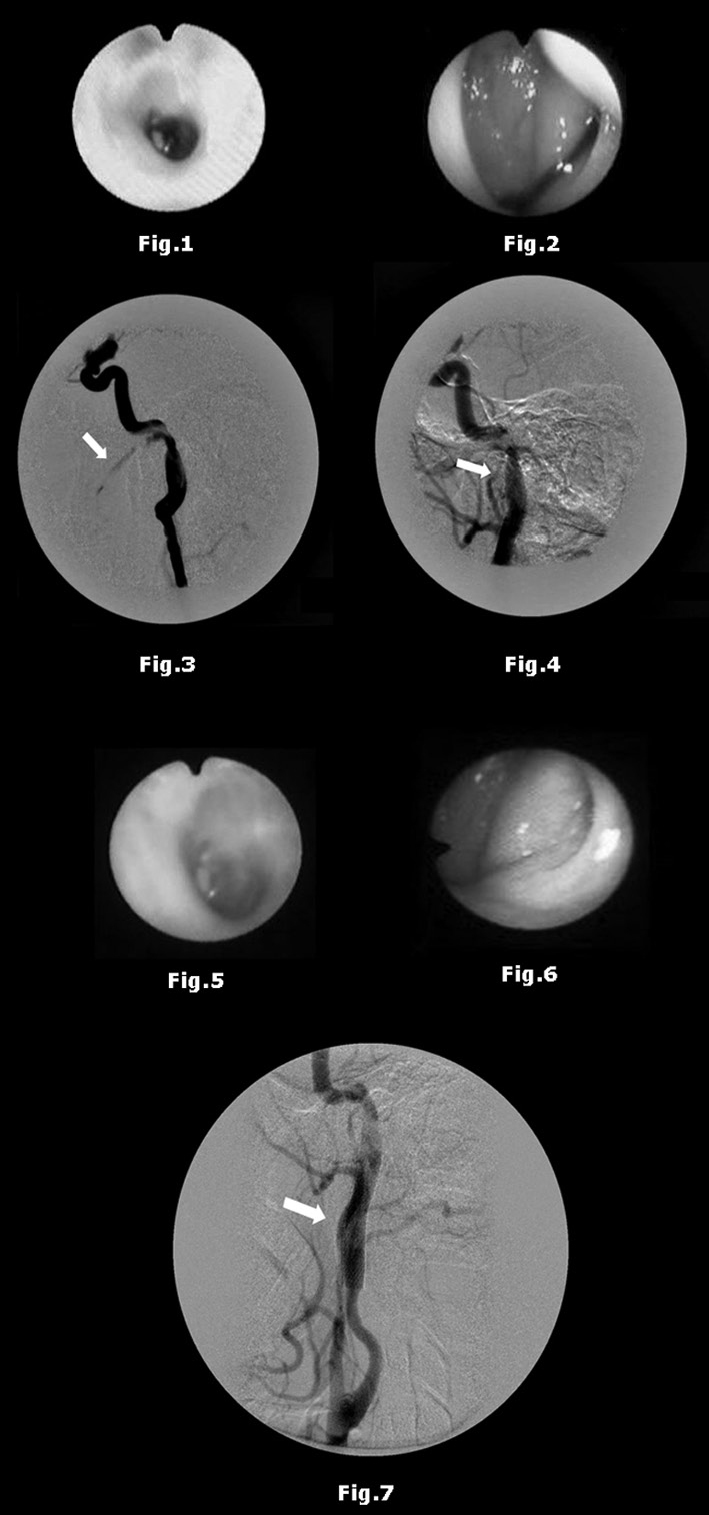
Nasal endoscopy 45 days after stenting – No signs of bleeding in the eustachian tube.

**Figure 7:**
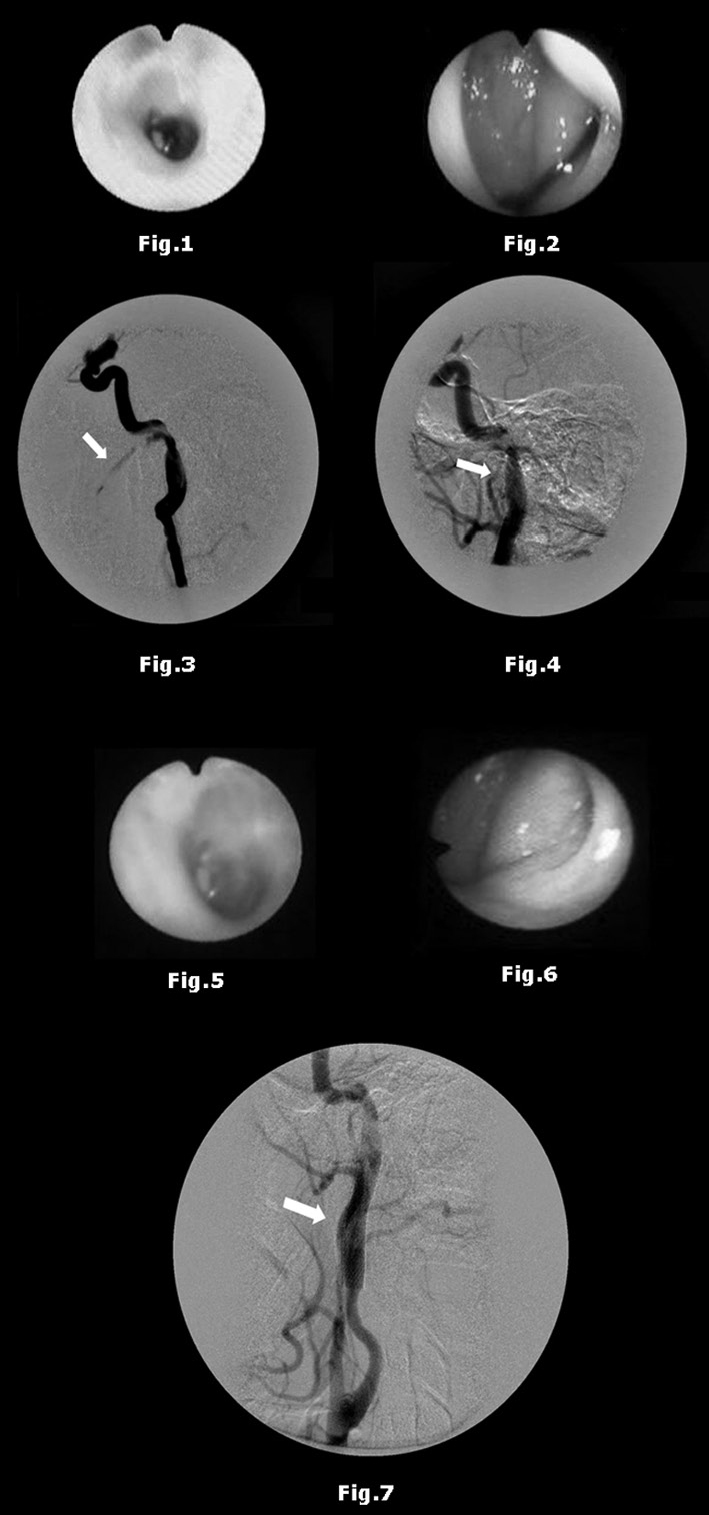
Angiogram 45 days after stenting – Stent patency and absence of signs of contrast spillover from the left ICA into the nasopharynx.

The etiology of her hypertension was studied while the patient was hospitalized. Pheochromocytoma, antiphospholipid syndrome, and renal artery stenosis were ruled out. Forty-five days into follow-up the patient had substantially improved her hearing acuity and had nearly no hemotympanum (Figure 5); nasal endoscopic examination showed no signs of bleeding through the eustachian tube (Figure 6); and her angiogram showed no signs of contrast spillover and patent stents (Figure 7). Ten months into follow-up the patient was neurologically asymptomatic and had no bleeding episodes. She complained of minor left ear hypacusis, while audiometry tests indicated mild left ear mixed hearing loss.

## DISCUSSION

The formation of an intracranial aneurysm or pseudoaneurysm in the ICA is related to blunt or penetrating trauma, surgery, endovascular procedure, inflammation, radiation^1^, ^4^, ^5^, vasculopathy, and genetic disorder. Manifestations such as cerebral ischemia or rupture and bleeding may occur months or even years after aneurysm or pseudoaneurysm installation^2^, ^4^. Digital subtraction angiography is still the gold standard for rupture diagnosis^1^, ^4^, although some authors have indicated CT angiography as the option for initial screening^4^. When the occlusion test is positive we are given the option of ligating the carotid artery, in a combined procedure in which the vessel is ligated in its neck and intracranial segments. Surgical treatment poses high levels of risk, and in our patient's case – as her occlusion test was negative – the most adequate approach is endovascular^3^, ^4^, ^6^. Anti-platelet drugs can be used for 30 days to prevent intra-stent thrombosis. When available, single-photon emission computerized tomography (SPECT) can be used to assess cerebral ischemia. SPECT was not used in our patient, although it was available at our hospital.

## CLOSING REMARKS

As a rare condition, epistaxis originating in the ICA is often diagnosed at later stages, thus worsening prognosis^3^, ^4^. Cases like the one presented in this description – a patient with poor overall health status, intense anemia, and transient ischemic episodes – call for broad multidisciplinary care so that early diagnosis and treatment can be established for ruptured intracranial segments of the ICA^1^, ^3^, ^4^. The endovascular stent approach was minimally invasive, effective, and safe for the presented case.
